# Disturbance of Oxidative Stress Parameters in Treatment-Resistant Bipolar Disorder and Their Association With Electroconvulsive Therapy Response

**DOI:** 10.1093/ijnp/pyaa003

**Published:** 2020-01-22

**Authors:** Qinyu Lv, Qiongyue Hu, Wenzhong Zhang, Xinxin Huang, Minghuan Zhu, Ruijie Geng, Xiaoyan Cheng, Chenxi Bao, Yingyi Wang, Chen Zhang, Yongguang He, Zezhi Li, Zhenghui Yi

**Affiliations:** 1 Shanghai Mental Health Center, Shanghai Jiao Tong University School of Medicine, Shanghai, China; 2 Qingdao Mental Health Center, Qingdao, China; 3 Department of Neurology, Ren Ji Hospital, Shanghai Jiao Tong University School of Medicine, Shanghai, China

**Keywords:** oxidative stress parameters, treatment-resistant bipolar disorder, electroconvulsive therapy

## Abstract

**Objective:**

Electroconvulsive therapy (ECT) is an effective option for treatment-resistant bipolar disorder (trBD). However, the mechanisms of its effect are unknown. Oxidative stress is thought to be involved in the underpinnings of BD. Our study is the first, to our knowledge, to report the association between notable oxidative stress parameters (superoxide dismutase [SOD], glutathione peroxidase [GSH-Px], catalase [CAT], and malondialdehyde [MDA]) levels and ECT response in trBD patients.

**Methods:**

A total 28 trBD patients and 49 controls were recruited. Six-week ECT and naturalistic follow-up were conducted. SOD, GSH-Px, CAT, and MDA levels were measured by enzyme-linked immunosorbent assay, and the 17-item Hamilton Depression Rating Scale and Young Mania Rating Scale were administered at baseline and the end of the 6th week. MANCOVA, ANCOVA, 2 × 2 ANCOVA, and a multiple regression model were conducted.

**Results:**

SOD levels were lower in both trBD mania and depression (*P = *.001; *P = *.001), while GSH-Px (*P = *.01; *P = *.001) and MDA (*P = *.001; *P = *.001) were higher in both trBD mania and depression compared with controls. CAT levels were positively associated with 17-item Hamilton Depression Rating Scale scores in trBD depression (r_adjusted_  = 0.83, *P = *.005). MDA levels in trBD decreased after 6 weeks of ECT (*P = *.001). Interestingly, MDA levels decreased in responders (*P = *.001) but not in nonresponders (*P > *.05).

**Conclusions:**

Our study indicates that decreased SOD could be a trait rather than a state in trBD. Oxidative stress levels are associated with illness severity and ECT response. This suggests that the mechanism of oxidative stress plays a crucial role in the pathophysiology of trBD.

**Significance Statement**

Although ECT is the effective option for trBD, the mechanisms of its effect are unknown. OxS is thought to be involved in the underpinnings of BD. We found SOD levels were lower in both trBD mania and depression, while GSH-Px and MDA were higher in both trBD mania and depression compared with controls. CAT levels were positively associated with HAMD-17 scores in patients with trBD depression. MDA levels in trBD decreased significantly after 6 weeks of ECT, especially in responders but not in nonresponders. These findings suggest that OxS may be involved in pathophysiology of trBD and ECT response.

## Introduction

Bipolar disorder (BD) is a chronic psychiatric disorder affecting approximately 0.4%–5.5% of the population worldwide; this disorder has high rates of recurrence and disability and imposes a large economic burden (Morgan et al., 2005;Bonnin et al., 2019). BD can carry a high risk of substance abuse, suicide, and mortality from comorbidities despite available pharmacological and psychosocial treatments (Schoepf and Heun, 2014; Hui Poon et al., 2015; Gold et al., 2018; Hansson et al., 2018). Furthermore, although mood stabilizers are available to treat BD, patients with unsatisfactory responses to pharmacological treatment are very common; this low responsiveness is known as treatment resistance. The definition of treatment-resistant BD (trBD) has been operationalized in different ways, with various requirements for the number and duration of treatment attempts (Dell’Osso et al., 2009; Pacchiarotti et al., 2009; Tondo et al., 2014; Hui Poon et al., 2015). The widely accepted definition encompasses patients who respond unsatisfactorily to at least 2 trials of dissimilar mood stabilizer regimens with adequate dose and duration within a specific phase of the illness (mania or depression), excluding patients who respond to pharmacological treatment but do not tolerate it (Sachs, 1996; Nierenberg et al., 2006; Sharma et al., 2008; Poon et al., 2012). To date, however, the underlying mechanisms of BD remain unknown; in particular, research on trBD is scarce.

Recent lines of evidence have indicated the mechanistic involvement of oxidative stress in the underpinnings of BD. Multiple studies have demonstrated that aberrations in the levels of oxidative stress species are associated with the onset and progression of several neuropsychiatric disorders, including BD (Andreazza et al., 2008, 2009; Berk et al., 2011; Kapczinski et al., 2011) and suicide attempts (Vargas et al., 2013). Furthermore, it is well established that increased oxidative stress in BD is usually correlated with exacerbated cognitive deficits (Vieta et al., 2013) and increased rates of medical comorbidities, especially metabolic disorders including obesity, a common comorbidity in BD (Birkenaes et al., 2007). Previous lines of evidence demonstrated that obesity or metabolic syndrome could complicate the symptoms and course of BD, indicating that oxidative stress plays a pivotal role in the pathophysiology of BD (Fagiolini et al., 2008; McIntyre et al., 2010; Leboyer et al., 2012; Hajek et al., 2016). In addition, several previous studies demonstrated that alterations in peripheral oxidative stress species were normalized by treatment (de Sousa et al., 2014; Tsai and Huang, 2015). Furthermore, N-acetylcysteine, an antioxidant supplement, alleviated the symptoms of BD (Berk et al., 2012, 2013).

The process of oxidative stress interacts with various neurobiological functions (including neuronal survival, growth, proliferation, differentiation, and plasticity) (Liu et al., 2017; Angelova and Abramov, 2018), mitochondrial function (Zorov et al., 2014), and immune response (including interaction and activation of immune cells, activation of the cascade of secondary autoimmune responses, and immunosuppression) (Yang et al., 2013), which are believed to be involved in the pathophysiology of BD.

The brain cells are vulnerable to exposure to oxidative stress, and they lack antioxidant defense systems to avoid excessive oxidative damage. Oxidative stress refers to an imbalance between oxidant and antioxidant factors in cellular metabolism, peroxidating membrane lipids and damaging organelles, proteins (including receptors, transcription factors, and enzymes) and DNA, ultimately causing cell damage. This state is considered to result from decreased antioxidant capacity or excessive production of oxidative stress species (Sies, 1991; Lenaz et al., 2000). Oxidative stress species are mainly generated by oxidative phosphorylation in the mitochondrial matrix during respiration (Adam-Vizi and Chinopoulos, 2006) and are regularly eliminated by cellular antioxidant species, including enzymatic and nonenzymatic antioxidants. Among these species, the most notably crucial parameters are antioxidant enzymes, including superoxide dismutase (SOD), glutathione peroxidase (GSH-Px), and catalase (CAT), and the end products and markers of lipid peroxidation, including malondialdehyde (MDA). Although several recent studies reported that these oxidative stress parameters were significantly altered in patients with BD, the results were inconsistent, and, to our knowledge, no study has focused on them in trBD.

Electroconvulsive therapy (ECT) is considered one of the effective treatment options for many psychotic disorders, especially treatment-resistant mood disorder and other psychiatric disorders (UK ECT Review Group 2003; Kellner et al., 2012; Schoeyen et al., 2015). However, the mechanisms of ECT remain unknown. Some previous studies found alterations in oxidant and antioxidant status after ECT in schizophrenia and mood disorders, but the results were inconsistent (Kartalci et al., 2011; Jorgensen et al., 2013; Genc et al., 2015). To the best of our knowledge, no study has reported the effect of ECT on oxidative mechanisms in trBD.

In the present study, we aimed to investigate (1) the levels of oxidative stress parameters (SOD, GSH-Px, CAT, and MDA) in trBD and their correlation with illness severity; (2) the effects of ECT on these 4 oxidative stress parameters in patients with trBD; and (3) whether they were associated with ECT response.

## Participants and Methods

### Participants

The protocol was reviewed and approved by the Institutional Review Board of Shanghai Mental Health Center. Written informed consent was obtained from each participant. All patients were recruited from Shanghai Mental Health Center from June 2014 to December 2016. All patients meeting the following inclusion criteria were recruited: (1) patients must be of Han Chinese ethnicity and must be 18 to 65 years old; (2) patients must satisfy the diagnostic criteria for BD according to the Diagnostic and Statistical Manual of Mental Disorders, Fourth Edition (DSM-IV); (3) patients must have 17-item Hamilton Depression Rating Scale (HAMD-17) scores ≥17 in BD depression or Young Mania Rating Scale (YMRS) scores ≥20 in BD mania; and (4) patients must meet the definition of trBD, that is, they have failed to respond to treatment with at least 2 well-recognized mood stabilizers, either as monotherapy or in combination, despite an adequate dose (concentration in serum) and duration (6 weeks for bipolar mania or 8 weeks for bipolar depression). The exclusion criteria eliminated the following candidates: (1) individuals with severe physical illness such as atherosclerosis, diabetes, hypertension, infection, or epilepsy; (2) women who were pregnant or planning to become pregnant during the study; (3) individuals with any other major Axis I disorder; (4) individuals with a history of ECT in the past 6 months; and (5) individuals who had taken antioxidant or vitamin supplements within 6 months before recruitment.

A total of 28 patients with trBD (13 males and 15 females) were recruited, including 14 with bipolar mania and 14 with bipolar depression; the patients had a mean age of 35.11 ± 15.78 years (mean ± SD) and a mean illness duration of 75.86 ± 85.03 months (mean ± SD) (demographic and clinical data are shown in Table 1).

**Table 1.** Demographic Characteristics in Patients With trBD and Healthy Controls

Variable	Patients (n = 28)	Controls (n = 49)	t or χ ^2^	*P*
Age (y, mean ± SD)	35.11 ± 15.78	21.12 ± 2.51	4.66	.001
Gender (male)	13 (46.43%)	20 (40.82%)	0.23	.63
BMI (mean ± SD)	23.44 ± 3.93	21.31 ± 2.71	2.81	.01
Age of onset (y, mean ± SD)	29.04 ± 13.48	–	–	–
Duration of illness (mo, mean ± SD)	75.86 ± 85.03	–	–	–
YMRS (trBD-M) (mean ± SD)	24.79 ± 2.89			
HAMD-17 (trBD-D) (mean ± SD)	21.86 ± 5.01	–	–	–

Abbreviations: BMI, body mass index; HAMD-17, Hamilton Depression Scale 17; trBD, treatment-resistant bipolar disorder; YMRS, Young’s Mania Rating Scale.

A total of 49 healthy controls (20 males and 29 females), with a mean age of 21.12 ± 2.51 years (mean ± SD), were recruited from the local community through advertisements. Individuals with any major Axis I disorders or family history of mental disorders were excluded. Any patients with severe cardiovascular disease, cerebrovascular disease, infections, cancer, diabetes, hypertension, or current pregnancy were excluded.

Medical history, physical examinations, laboratory tests, electrocardiography, and electroencephalography were obtained from each of the patients and healthy controls before recruitment into this study. Any patients with drug or alcohol abuse/dependence were identified by self-reported drug use and medical records were excluded.

### Demographic Information Collection and Clinical Assessments

We designed a questionnaire reflecting the socio-demographic profile and the physical and psychological situation of the patient, and we administered this questionnaire to all participants at baseline. Patients were diagnosed by 2 experienced psychiatrists based on the Structured Clinical Interview for DSM-IV Axis I Disorders, Patient Version, as in our previous report (Li et al., 2017). The severity of symptoms was assessed by administering the HAMD-17 and YMRS at baseline and at the end of 6-week ECT. Response to ECT in bipolar depression was defined as a HAMD-17 score reduction of ≥50% or a HAMD-17 score = 7. Response to ECT in bipolar mania was defined as a YMRS score reduction rate of ≥50% or a YMRS score = 12 (Grof et al., 2002; Lv et al., 2019).

All members of the research staff were trained in the use of the Structured Clinical Interview for DSM-IV Axis I Disorders, Patient Version, HAMD-17, and YMRS before the study. Inter-rater reliability was maintained at more than 0.80 for the HAMD-17 and YMRS, as in our previous report (Li et al., 2014).

### ECT Administration and Naturalistic Follow-up

Patients with trBD were treated with modified ECT for 6 weeks (2–3 times a week for up to 12 sessions). The ECT treatments were performed using the Thymatron System IV device (Somatics, Lake Bluff, IL) according to the following standardized protocol. Patients fasted for 6 hours before treatment and received successive i.v. injections of atropine (0.5 mg), propofol (1.5~2.0 mg/kg), and succinylcholine chloride (0.8~1.0 mg/kg) from 8:30 am to 9:30 am. After approximately 90 seconds of muscle relaxation, modified ECT was administered. Stimulation electrodes were placed bilaterally on the temples. The pulse duration and width were set to 3–8 milliseconds and 0.5 milliseconds, respectively. The measured static resistance values ranged from 250 to 1500 Ω. The amount of stimulation was set according to age and drug use, and the doses of anesthetics and muscle relaxants were calculated according to body weight. The stimulus intensity was determined by the titrated strategy (Kellner et al., 2010). In briefly, stimulus energy value was set based on age minus 5, and if patients were treated with diazepam and antiepileptic drugs, stimulus energy value plus 5. ECT was performed by 1 deputy chief psychiatrist with more than 10 years of experience in conducting the procedure. The ECT parameters were rigorously checked before treatment. During ECT sessions, previous mood stabilizers and antipsychotics were permitted, while anticonvulsants were withdrawn on the day with ECT. Among 28 trBD patients, 5 patients were drug free, 2 patients were taking lithium combined with antipsychotics, 1 patient was on valproate monotherapy, 4 patients were on antidepressant monotherapy, 4 patients were on antidepressants combined with antipsychotics, 4 patients were on valproate combined with antidepressants, 3 patients were on valproate combined with antipsychotics, 3 patients were on valproate combined with antidepressants and antipsychotics, and 2 patients were on lithium combined with valproate and antipsychotics. Brief use of benzodiazepines was permitted for patients with insomnia or anxiety.

### Sample Preparation and Measurement of Plasma Oxidative Stress Parameters

A 5-mL volume of peripheral venous blood was collected from each of the fasting patients from 7:00 am to 9:00 am before ECT at baseline and at the end of the 6th week. Similarly, a 5-mL volume of peripheral venous blood was collected from the healthy controls from 7:00 am to 9:00 am after recruitment. The plasma was subsequently isolated, aliquoted, and frozen at −80°C. SOD, GSH-Px, CAT, and MDA levels were measured in duplicate with an enzyme-linked immunosorbent assay according to the manufacturer’s protocol (R&D Systems, Inc., Minneapolis, MN). The colorimetric method was used to react on the enzyme plate to determine the optical density value. All the tests were carried out by special technicians of Shanghai Mental Health Center, and all samples were tested at the same time. Furthermore, random sample measurements were repeated to confirm the reproducibility of the assay, and the inter-assay coefficient of variation was 5.18%. The experimenters were blinded to all the clinical data.

### Statistical Analyses

Means and SDs were used to describe continuous variables, while numbers and percentages were computed for categorical variables. Student’s *t* test and paired *t* test were used for continuous variables, and chi-squared test was used for categorical variables. A 1-sample Kolmogorov-Smirnov test was used to test the normality of the distribution.

To investigate the difference in each oxidative stress parameter between patients and controls, we first conducted MANCOVA, considering these 4 oxidative stress parameters were multiple continuous dependent variables and they might have interact with each other. Thus, it could lead to a type I error without this initial test. In the MANCOVA model, the overall *P* value was calculated, with all oxidative stress parameters as the dependent variables, diagnosis as the fixed variable, and age and body mass index (BMI) as the covariates. ANCOVA was then performed with each oxidative stress parameter as the dependent variable, diagnosis as the fixed variable, and age and BMI as the covariates. Bonferroni corrections were applied to adjust for multiple testing.

Partial correlation and multiple variable regression models were used to detect the association between illness severity and each oxidative stress parameter, adjusting for age, gender, BMI, smoking, age of onset, and duration of illness.

To detect the effects of different pharmacological treatments on each oxidative stress parameter, we conducted a 2 × 2 (treatment × time) ANCOVA. The main effects of treatment (different treatment strategies) and time (baseline and 6 weeks later) and the treatment × time interaction effect were assessed.

To evaluate the difference in each oxidative stress parameter before and after ECT treatment, we used a paired-sample *t* test. Further, to examine whether the change of OxS parameters levels could predict the ECT response, logistic regression analysis (backward: Wald) was conducted, with response as an independent variable (nonresponse = 0, response = 1), with age, gender, BMI, smoking, age of onset, and duration of illness as covariates. The software programs PASW Statistics 18.0 (SPSS Inc., Chicago, IL) and GraphPad Prism 7.0 (GraphPad Software, Inc., San Diego, CA) were used to perform statistical analysis and generate graphs. All *P* values were 2-tailed, and the significance was set at .05.

## Results

Demographic and clinical characteristics of patients and controls are detailed in Table 1. There was no significant difference in gender (χ ^2^  = 0.23, *P = *.63), whereas there were significant differences in age and BMI between patients and controls (t = 4.66, *P = *.001; t = 2.81, *P = *.01).

### Oxidative Stress Parameters in Patients and Healthy Controls at Baseline

**Figure 1.**
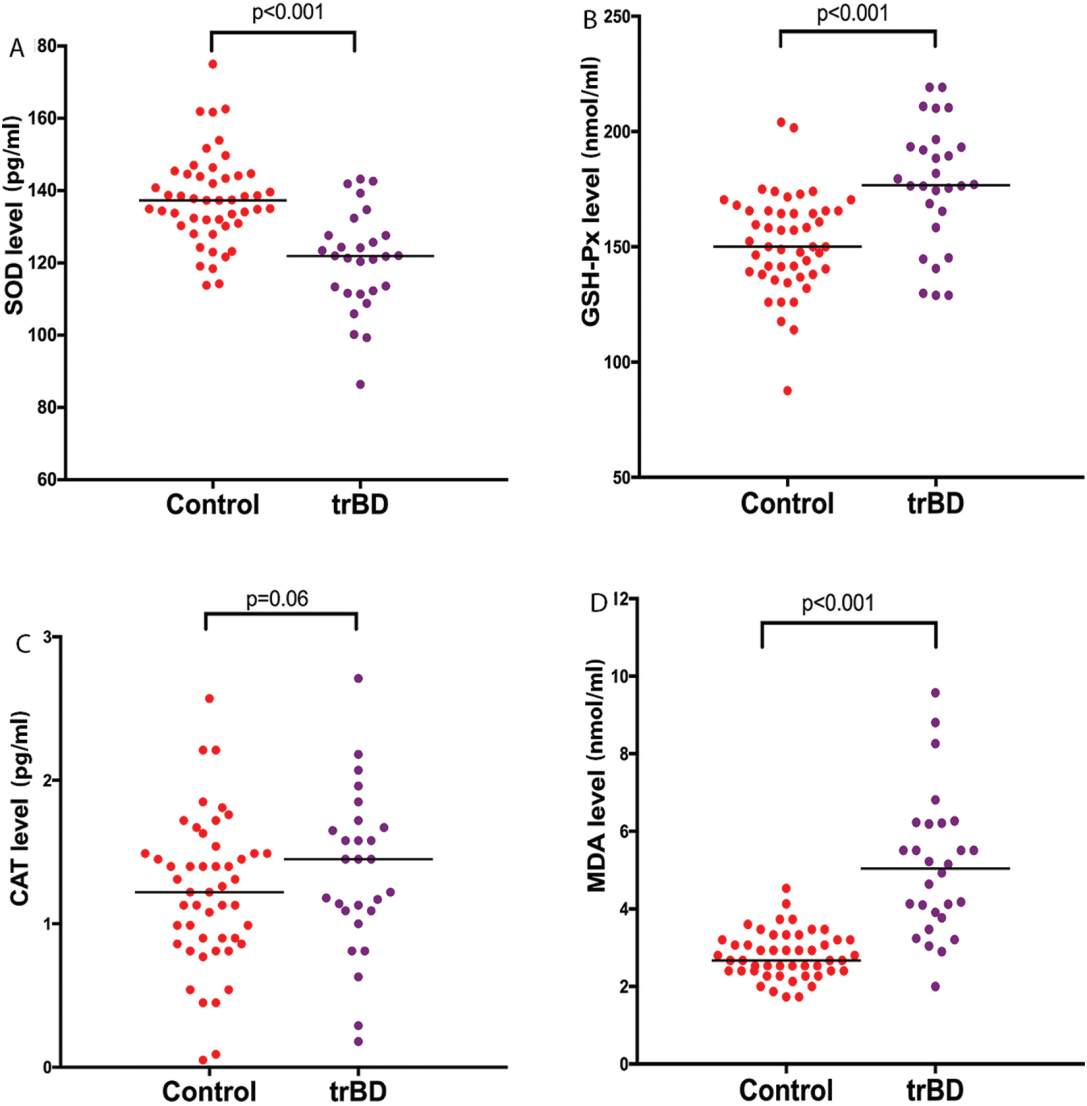
Oxidative stress parameters in patients and healthy controls at baseline. ANCOVA was applied to compare oxidative stress parameters (SOD, superoxide dismutase; GSH-Px, glutathione peroxidase; CAT, catalase; MDA, malondialdehyde) between patients and controls adjusting for age. There were significant differences in the levels of SOD, GSH-Px, and MDA between patients and controls (all *P = *.001), whereas CAT levels were not significantly different (*P > *.05). trBD, treatment-resistant bipolar disorder.

MANCOVA analysis demonstrated significant differences in all oxidative stress parameter levels between patients and controls (Wilks’ lambda F = 23.21, *P = *.0001). Then, ANCOVA was applied to compare oxidative stress parameters (SOD, GSH-Px, CAT, MDA) between patients and controls, adjusting for age and BMI. As shown in Figure 1, SOD levels were lower in trBD patients than in controls, while GSH-Px and MDA levels were higher in trBD patients than in controls (all *P = *.001). There was no difference in CAT levels between trBD patients and controls (F = 2.99, *P = *.06).

**Figure 2.**
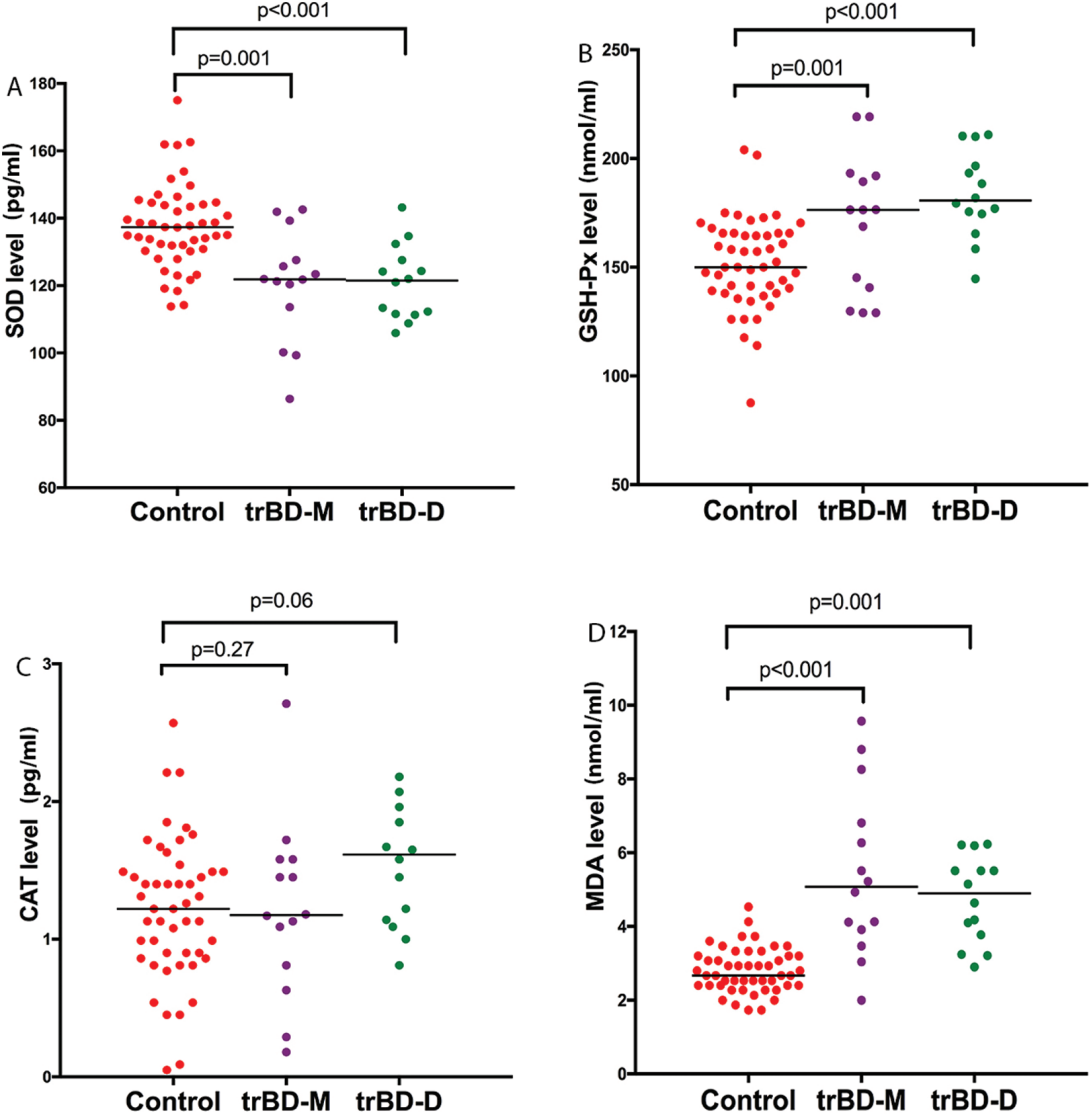
Oxidative stress parameters among treatment-resistant manic bipolar disorder (BD) patients, treatment-resistant depressive BD patients, and healthy controls at baseline. ANCOVA was applied to compare oxidative stress parameters (SOD, superoxide dismutase; GSH-Px, glutathione peroxidase; CAT, catalase; MDA, malondialdehyde) among the 3 groups. Bonferroni corrections were applied to adjust for multiple testing. trBD-D, treatment-resistant bipolar disorder depression; trBD-M, treatment-resistant bipolar disorder mania.

As shown in Figure 2, when trBD patients were divided into manic and depressive groups, ANCOVA adjusting for age and BMI (Bonferroni corrections) showed that (1) SOD levels were lower in both the manic and depressive groups than in the control groups (F = 12.46, *P = *.001; F = 13.88, *P = *.001, respectively), whereas the manic and depressive groups did not differ (F = 0.05, *P = *.94); (2) GSH-Px levels were higher in both the manic and depressive groups than in the control group (F = 6.56, *P = *.01; F = 18.88, *P = *.001, respectively), whereas the manic and depressive groups did not differ (F = 1.92, *P = *.17); (3) MDA levels were higher in both the manic and depressive groups than in the healthy control group (F = 13.65, *P = *.001; F = 21.27, *P = *.001, respectively), whereas the manic and depressive groups did not differ (F = 1.26, *P = *.27); (4) there was no significant difference in CAT levels among the manic, depressive, and control groups (F = 1.98, *P = *.15).

### Association Between Oxidative Stress Parameters and Illness Severity in Patients at Baseline

**Figure 3.**
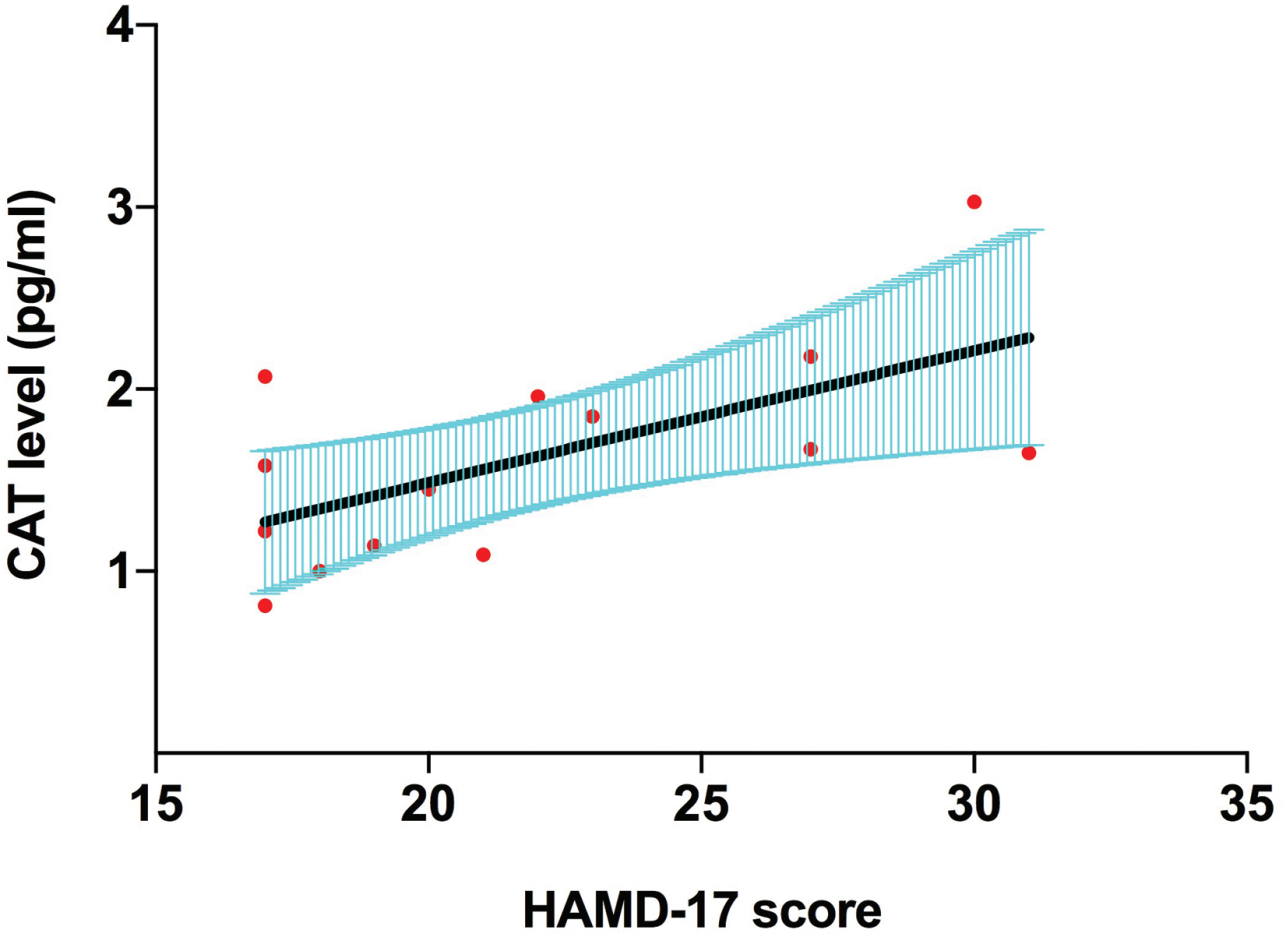
The association between catalase (CAT) levels and illness severity in patients with treatment-resistant bipolar disorder (tr-BD) depression at baseline. CAT levels were positively associated with 17-item Hamilton Depression Rating Scale (HAMD-17) scores in patients with trBD depression (adjusted r = 0.83, *P = *.005).

To clarify the potential effects of confounding factors, partial correlation analysis was used to examine the association between each oxidative stress parameter and illness severity, adjusting for age, gender, BMI, smoking, age at onset, and duration of illness. As shown in Figure 3, CAT levels were positively associated with HAMD-17 scores in patients with trBD depression (adjusted r = 0.83, *P = *.005), while SOD, GSH-Px, and MDA levels were not associated with HAMD-17 scores in patients with trBD depression (SOD, adjusted r = 0.46, *P = *.22; GSH-Px, adjusted r = 0.44, *P = *.23; MDA, adjusted r = 0.16, *P = *.69). There was no association of each OxS parameter with YMRS score in trBD mania (SOD: adjusted r = 0.26, *P = *.51; GSH-Px: adjusted r = 0.47, *P = *.20; CAT: adjusted r = 0.16, *P = *.68; MDA: adjusted r = 0.41, *P = *.28).

We used a stepwise multiple regression model to further investigate the association between each oxidative stress parameter and illness severity, adjusting for age, gender, BMI, smoking, age at onset, and duration of illness. CAT levels were positively associated with HAMD-17 scores in patients with trBD depression (CAT, B = 0.12, t = 3.99, *P = *.005), while SOD, GSH-Px, and MDA levels were not associated with HAMD-17 scores in patients with trBD depression (SOD: B = 1.20, t = 1.36, *P = *.22; GSH-Px: B = 2.29, t = 1.31, *P = *.23; MDA: B = 0.03, t = 0.42, *P = *.69). There were associations of each OxS parameter with YMRS score in trBD mania (SOD: B = 0.82, t = 0.70, *P = *.51; GSH-Px: B = 3.32, t = 1.40, *P = *.20; CAT: B = 0.02, t = 0.43, *P = *.68; MDA: B = 0.12, t = 1.18, *P = *.28).

### The Effect of Different Treatment Strategies on Oxidative Stress Parameters

In the present study, different treatment strategies including (1) 5 drug-free patients; (2) 2 patients with combination of lithium and antipsychotics; (3) 1 patient with valproate monotherapy; (4) 4 patients with antidepressant monotherapy; (5) 4 patients with a combination of antidepressants and antipsychotics; (6) 4 patients with a combination of valproate and antidepressants; (7) 3 patients with a combination of valproate and antipsychotics; (8) 3 patients with a combination of valproate, antidepressants, and antipsychotics; and (9) 2 patients with a combination of lithium, valproate, and antipsychotics. The 2 × 2 (treatment × time) ANCOVA showed that there was no significant effect of different treatment strategies on the levels of CAT (F = 1.15, *P = *.38), MDA (F = 0.79, *P = *.62), GSH-Px (F = 1.37, *P = *.27), or SOD (F = 0.56, *P = *.80). There was also no significant treatment × time interaction effect on the levels of CAT (F = 0.63, *P = *.74), MDA (F = 0.49, *P = *.85), GSH-Px (F = 1.56, *P = *.20), or SOD (F = 0.98, *P = *.48).

### Effect of 6-Week ECT on Oxidative Stress Parameters

In patients with trBD, both CAT and MDA levels decreased significantly compared with baseline after 6 weeks of ECT (CAT: 1.42 ± 0.64 vs 1.08 ± 0.57, t = 2.56, *P = *.02; MDA: 5.09 ± 1.80 vs 3.84 ± 1.12, t = 4.05, *P = *.001), but the CAT levels did not change after Bonferroni correction for multiple tests. No differences in SOD or GSH-Px levels were found after 6 weeks of ECT compared with pretreatment levels (SOD: 123.85 ± 15.34 vs 120.65 ± 13.65, t = 1.01, *P = *.32; GSH-Px: 176.82 ± 26.63 vs 178.70 ± 22.38, t = 0.53, *P = *.60).

**Figure 4.**
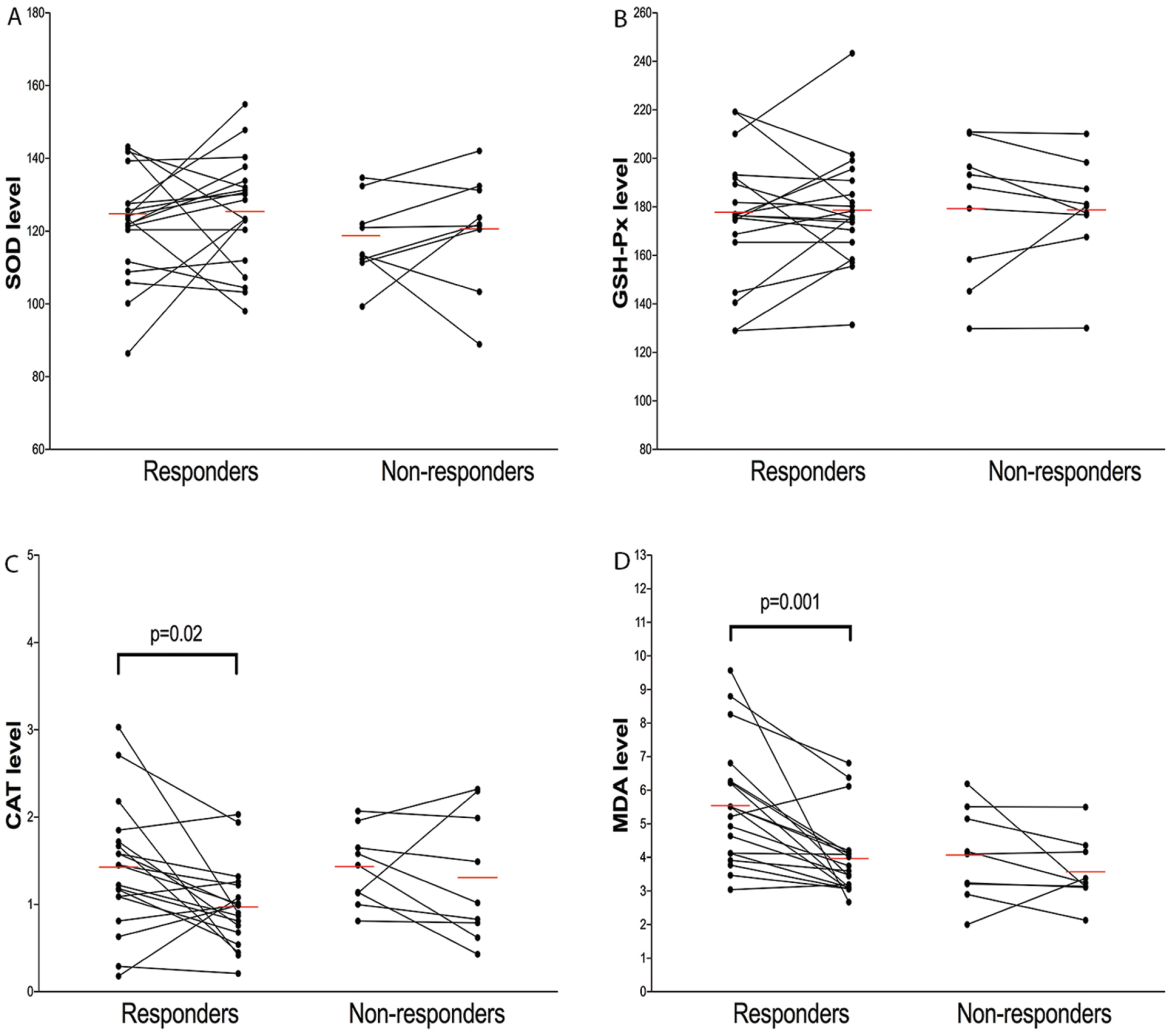
The effect of 6-week electroconvulsive therapy (ECT) treatment on oxidative stress parameters. Catalase (CAT) and malondialdehyde (MDA) levels decreased in responders after 6 weeks of ECT (*P = *.02 and *P = *.001, respectively), but the CAT levels did not change after Bonferroni correction for multiple tests. Red line represents the mean level of each parameter. Responders, patients who responded to ECT; nonresponders, patients who failed to respond to ECT.

After 6 weeks of ECT, 19 patients were determined to be responders, while 9 patients were nonresponders (comprising 2 with trBD mania and 7 with trBD depression). When patients were divided into responders and nonresponders, there were no differences in the use of mood stabilizers or antipsychotics between responders and nonresponders (χ ^2^ = 0.02, *P = *.90). As shown in Figure 4, we found that both CAT and MDA levels decreased in responders after 6 weeks of ECT (CAT: 1.42 ± 0.72 vs 0.97 ± 0.46, t = 2.66, *P = *.02; MDA: 5.57 ± 1.81 vs 3.97 ± 1.19, t = 4.11, *P = *.001), but the CAT levels did not change after Bonferroni correction for multiple tests. CAT and MDA levels did not decrease in nonresponders (CAT: 1.42 ± 0.43 vs 1.31 ± 0.73, t = 0.55, *P = *.60; MDA: 4.05 ± 1.36 vs 3.57 ± 0.97, t = 1.21, *P = *.26). Neither SOD nor GSH-Px levels were altered in responders (SOD: 122.01 ± 14.80 vs 125.37 ± 15.25, t = 0.80, *P = *.43; GSH-Px: 175.72 ± 26.33 vs 178.66 ± 23.05, t = 0.63, *P = *.54) or in nonresponders (SOD: 117.79 ± 11.05 vs 120.62 ± 15.93, t = 0.60, *P = *.57; GSH-Px: 179.15 ± 28.70 vs 178.78 ± 22.24, t = 0.07, *P = *.95). Logistic regression analysis showed that the changes of SOD, GSH-Px, CAT, and MDA were not associated with ECT response (*P = *.67, *P = *.83, *P = *.13, and *P = *.63, respectively).

## Discussion

Oxidative stress has been implicated in the pathophysiology of BD (Andreazza et al., 2008, 2009); however, the levels of oxidative stress parameters in trBD and the effects of ECT on them were uninvestigated. To the best of our knowledge, this is the first study to investigate the levels of oxidative stress parameters (SOD, GSH-Px, CAT, and MDA) in trBD and the effect of ECT on oxidative metabolism in these patients. In the present study, we found the following: (1) SOD levels were lower in both trBD mania and trBD depression than in controls, while GSH-Px and MDA levels were higher in both trBD mania and trBD depression than in controls; (2) CAT levels were positively associated with HAMD-17 scores in patients with trBD depression; and (3) MDA levels in trBD decreased significantly after 6 weeks of ECT. Interestingly, we found that MDA levels decreased in responders after 6 weeks of ECT but did not decrease in nonresponders.

Although results concerning SOD in BD have been inconsistent (Andreazza et al., 2008; Brown et al., 2014), we found that SOD levels were lower in trBD (both manic and depressive episodes) than in healthy controls, but the levels did not change after 6 weeks of ECT, even in ECT responders, suggesting that they could be a trait rather than a state in trBD. Reduced SOD levels in BD indicate dysfunction in cell membrane repair mechanisms (Reddy et al., 1991). A previous study also demonstrated that antidepressants and ECT had no effects on SOD levels, indicating that SOD activity may be independent of specific treatments (Selek et al., 2008). These results imply that a decrease in the antioxidative function of SOD may play a critical role in the pathogenesis of trBD.

Some previous studies showed that GSH-Px levels decreased in BD patients, indicating reduced antioxidative mechanisms in BD (Raffa et al., 2012; Rosa et al., 2014; Tsai and Huang, 2015; Nucifora et al., 2017). Other studies demonstrated increased GSH-Px levels in BD. They found that GSH-Px levels were correlated with the duration of BD and increased in the late stage of BD, suggesting that the cumulative effect of ongoing oxidative stress along with increased duration of illness could cause increased GSH-Px levels as a compensatory mechanism to avoid further oxidative damage in BD (Andreazza et al., 2009). In the present study, we found that GSH-Px levels increased in trBD (both manic and depressive episodes); in most cases, the duration of illness was more than 12 months (85.71%), and the patients had received mood stabilizers for a period of time. Our data also indicated that increased GSH-Px activity in trBD might be the result of a compensatory response to chronic oxidative stress. Although compensatory antioxidant mechanisms could play an important role in the progression of BD, it is likely that such compensatory mechanisms are only partially effective (Andreazza et al., 2009). For example, de Sousa et al. demonstrated that glutathione peroxidase and CAT levels increased in BD depression (de Sousa et al., 2014). We also found that CAT levels were positively associated with HAMD-17 scores at baseline in trBD depression and decreased after 6 weeks of ECT, which suggested that CAT levels could indicate the severity of trBD depression. CAT is one of the most important antioxidants that can also reduce H_2_O_2_ to water and molecular oxygen. In line with the compensatory mechanism of GSH-Px, we suggested more severe symptoms accompanied with higher CAT levels, avoiding further oxidative damage in trBD depression.

MDA, a marker of oxidative stress, results from lipid peroxidation of polyunsaturated fatty acids. Most previous studies reported that MDA levels and lipid peroxidation increased in patients with BD (Yumru et al., 2009; Kartalci et al., 2011; Brown et al., 2014) and significantly decreased with lithium treatment (Bengesser et al., 2016). In addition, a previous study demonstrated that serum MDA levels were significantly reduced after the ninth ECT session in patients with schizophrenia (Kartalci et al., 2011), and an animal study supported the idea that lipid peroxidation decreased in the hippocampus, cerebellum, and striatum after single or multiple electroconvulsive shock (Barichello et al., 2004). In the present study, we found that MDA levels increased in trBD (both manic and depressive episodes) prior to treatment. Furthermore, we found that MDA levels decreased in responders but not in nonresponders after 6 weeks of ECT. The results indicated that the level of lipid peroxidation decrease contributed to ECT efficiency.

Previous animal studies have demonstrated that electroconvulsive shock (ECS) (the animal equivalent of ECT) had positive effects on oxidative stress parameters in the brain. For example, Barichello et al. showed that single electroconvulsive and multiple ECS administration could downregulate the lipid peroxidation levels, whereas it upregulated SOD and CAT levels in various brain domains of rats (Barichello et al., 2004). However, the underlying mechanisms of the effect of ECT on peripheral oxidative stress markers are still unclear. There are 2 potential pathways which may be involved in the mechanisms of the effect of ECT on oxidative stress status. Previous evidence demonstrated that ECT could affect NMDA and the neuroinflammation pathway. For example, Dong et al. indicated that ECT could increase the NMDA receptor expression but decrease this receptor stimulation threshold in the rat brain (Dong et al., 2010). Pfleiderer et al. showed that ECT could reverse glutamatergic deficit in left anterior cingulum of the depressed patients (Pfleiderer et al., 2003). Shibasaki et al. showed that reduction of MMP-9 levels were associated with ECT response in patients with depression (Shibasaki et al., 2018). On the other hand, NMDA and neuroinflammation pathway had reciprocal interaction with the oxidative stress system (Kamat et al., 2016; Steullet et al., 2016).

However, it is still unknown whether such alteration attributed to the direct effect of the ECT on the oxidative stress status or whether it is secondary to alleviation of symptoms. Thus, further study should be conducted to investigate the potential mechanisms. It should be noted that the oxidative stress parameters levels were measured from the peripheral blood, which might not reflect their levels in the brain. Until now, it remained unclear whether peripheral levels of oxidative stress parallel those in the brain. Previous review indicated that the neurons in the brain are sensitive to oxidative stress exposure, and peripheral oxidative stress can affect the activation of oxidative stress in neurons (Maciejczyk et al., 2019). However, the results were not consistent. For example, Xin et al. conducted a magnetic resonance spectroscopy (MRS) study and showed that low GSH levels in brain correlated with low peripheral oxidative stress status in healthy controls but with high oxidative stress status in patients with early psychosis (Xin et al., 2016). Another recent MRS study conducted by Silva et al. demonstrated that there was no association between brain GSH levels and peripheral GPx activity in both healthy controls and patients with clinical high risk for psychosis (Da Silva et al., 2018). Further MRS study should be conducted on the effects of ECT on brain oxidative stress parameters.

The present study has some limitations that should be considered. First, our study used a naturalistic observation design that did not allow complete control over confounding factors. At baseline, trBD patients had already received mood stabilizers for a period of time, which could affect the oxidative parameters, although previous studies indicated that some oxidant species (e.g., SOD) might be independent of specific treatments such as antidepressants and ECT (Selek et al., 2008). This complex situation was especially difficult to control in a real-world study, and further prospective interventional cohort studies should be conducted to validate our results. Second, the oxidative stress parameter levels were measured from the peripheral blood, and further MRS study should be conducted together to detect oxidative stress status in the brain of the patients with BD. Third, the difference in age between trBD and control has significance, even though it was adjusted for as covariate. Fourth, in this study, substance use was identified by self-reported drug use and medical records, rather than all participants being examined by urine drug tests. Lastly, we used enzyme-linked immunosorbent assay method to detect the oxidative stress parameters rather than liquid chromatography-mass spectrometry, which has higher specificity and detection sensitivity.

Despite some limitations, our study indicated that decreased SOD levels in trBD could be a trait rather than a state. GSH-Px levels increased in trBD. CAT levels were positively associated with illness severity in depressive trBD patients. Further, MDA levels decreased only in responders to ECT, which suggested that decrease in MDA was involved in ECT efficiency. Our results suggest that the mechanism of oxidative stress might play a crucial role in the pathophysiology of trBD.

## Statement of Interest

None.
